# What Is Hidden behind Amputation? Quanti-Qualitative Systematic Review on Psychological Adjustment and Quality of Life in Lower Limb Amputees for Non-Traumatic Reasons

**DOI:** 10.3390/healthcare11111661

**Published:** 2023-06-05

**Authors:** Laura Calabrese, Marina Maffoni, Valeria Torlaschi, Antonia Pierobon

**Affiliations:** Istituti Clinici Scientifici Maugeri IRCCS, Psychology Unit of Montescano Institute, 27040 Montescano, Italy

**Keywords:** amputation, amputee, lower limb, psychological adjustment, well-being, health-related quality of life, anxiety, depression

## Abstract

Objective: This systematic review aims to investigate Quality of Life (QoL)/Health Related Quality of Life (HRQoL) and psychological adjustment in non-traumatic lower limb amputees (LLA). Methods: PubMed, Scopus, and Web of Science databases were used for the literature search. Studies were read and analysed using the (Preferred Reporting Items for Systematic Reviews and Meta-Analyses) PRISMA statement procedure. Results: The literature search retrieved 1268 studies, of which 52 were included in the systematic review. Overall, psychological adjustment, especially depression with or without anxiety symptoms, influences the QoL/HRQoL in this clinical population. Other factors influencing QoL/HRQoL include subjective characteristics, physical aspects, the cause and level of the amputation, relational aspects, social support, and the doctor-patient relationship. In addition, the patient’s emotional-motivational status, depression and/or anxiety symptoms, and acceptance play a key role in the subsequent rehabilitation process. Conclusions: In LLA patients, psychological adjustment is a complex and multifaceted process, and QoL/HRQoL may be influenced by various factors. Shedding light on these issues may provide useful suggestions for promoting clinical and rehabilitative interventions that may be tailored and effective in this clinical population.

## 1. Introduction

Amputation is the surgical removal, or accidental loss, that involves the elimination of part or all of a limb [1]. It therefore refers to an acquired condition caused by injury, disease, or surgery [2]. This procedure can be used when arterial reconstruction surgery is not technically possible, has failed, or when the limb has lost its function. For example, amputation can be the result of various conditions such as peripheral vascular disease, trauma, malignancy, metabolic disease, and infection [2]. According to the literature, lower limb amputation (LLA) represents 80–85% of all amputations and is mainly due to vascular diseases such as diabetes mellitus, atherosclerosis, and Buerger’s disease [3].

The choice of amputation level is based on the patient’s postoperative function and the best possible primary wound healing [4]. In this sense, the concept of lower limb amputation can encompass different types of amputation, allowing different issues and treatments to be considered in order to provide comprehensive patient care.

### 1.1. Physical and Psychological Well-Being

The literature and clinical experience show that amputation has a significant impact on physical and psychological well-being, resulting in various life changes [5]. In fact, one of the most important consequences is the functional limitation, which, if not addressed, may lead to permanent disability, and, in turn, may be experienced as a traumatising experience [6]. The loss of a limb is therefore a significant experience that causes disruption in several areas of the person’s life: change in mobility [7], participation in social activities [7], possibility of returning to work [8], and the patient’s mood [9].

In addition, amputation has an impact on psychological well-being, vulnerability, and the definition and redefinition of identity [7]. The loss of a limb also affects the perception of body image. Specifically, increasing levels of amputation are associated with greater discomfort with body image [5]. In addition, amputation is associated with anxiety, depression, social isolation, and pain [10]; it even predicts a change in leisure activities [11] and social position within the community [7]. 

Overall, the loss of a body part may lead to a period of grief that requires time for readjustment. During this period, physical, functional, and psychological problems related to the difficulty of adjustment may occur [7]. 

In summary, there are several factors that influence the outcome of this period, and various studies have focused on physical factors, especially the age of the amputee [12], the level of amputation, and the presence of comorbidities [13].

### 1.2. Rehabilitation after Amputation

After amputation, patients begin a rehabilitation process that includes adapting to various physical and psychosocial challenges [14].

Factors that have a positive impact on rehabilitation include the ability to perform activities prior to amputation, no or little delay in admission to a rehabilitation centre, patient motivation, and good communication with the rehabilitation team. In this regard, the presence of a multidisciplinary team appears to promote a positive outcome for the amputee [15]. The team, working in a coordinated manner, aims to bring the amputee to the highest level of functional recovery in relation to his or her potential [7]. This can be achieved through the use of prostheses, which counteract the negative effects of amputation by helping to reduce comorbidity [16]. Prosthetics can be seen as a relevant indicator of quality of care and quality of life. Patient satisfaction plays a key role in the recovery of mobility and adherence to treatment [7]. However, it should be emphasised that more than 40–60% of amputees are not satisfied with their prostheses. Failure to overcome the initial problems caused by the amputation difficulties in fitting and wearing the prosthesis may lead to sporadic use of it or even complete rejection [13,15].

### 1.3. Quality of Life and Psychological Adjustment

Amputation is therefore an intervention that affects the whole of the amputee’s existence and, in turn, his or her Quality of Life in general terms (QoL) and Health-Related Quality of Life (HRQoL) in particular [17].

Specifically in amputees, QoL/HRQoL is associated with several aspects such as prosthesis use [7], presence of pain [18], occupational reintegration [18], level of amputation [18], and social support [19]. Studying QoL/HRQoL in amputees remains complex, hampered by methodological issues such as heterogeneity concerning samples and measures [20].

Psychological adjustment is the process activated in response to illness, a disabling event, and its treatment [21], and is a key factor to consider in amputees. Individuals must come to terms with the new physical condition, the various psychological consequences, and the underlying causes of the amputation [22]. There is therefore a large interindividual variability in response to the same adverse event, specifically amputation. For example, different reactions may depend on the patient’s gender, age, personality factors, social support, experience of pain, cause of amputation, and time since surgery [23]. However, there are some common reactions, which include anxiety, reduced quality of life, depressed mood, pain, body image concerns, dysfunctional coping strategies, and difficulties in social interactions [13,24]. Amputees therefore experience a range of complex psychological reactions. Emotional difficulties may interfere with functional recovery and rehabilitation itself. Among these, depression remains the most common response, negatively affecting psychological and physical adjustment [1]. The presence of postoperative depressive symptoms is common [25], but these appear to decrease over time [26]. Anxiety is also associated with the fear of losing physical function and mobility. Another aspect that influences the outcome of psychological adjustment is the coping strategy adopted. Coping refers to the way in which adverse situations are managed and the responses that are used to deal with them. Specifically, people who tend to focus on a problem-solving approach are associated with a positive psychosocial adjustment outcome [27] and increased functionality; conversely, this coping style seems to be negatively associated with depression [22]. 

Thus, considering how many aspects may influence the well-being of LLA patients, this systematic review aims to provide an overview of the current state of the literature on quality of life and psychological adjustment in non-traumatic LLA patients, highlighting risk and protective factors that have not yet been studied in depth.

## 2. Materials and Methods

A systematic review was conducted to identify articles related to psychological adjustment and QoL/HRQoL in lower limb amputees. Data were analysed and reported according to the international PRISMA (Preferred Reporting Items for Systematic Reviews and Meta-Analyses) guidelines [28].

### 2.1. Search Strategy and Data Extraction

Three publicly accessible databases (PubMed, Scopus, and Web of Science) were used for the electronic literature search using the following terms: (“lower extremity amput*” OR “lower limb amput*”) AND (psychological OR acceptance OR adherence OR compliance OR “health-related quality of life”). Articles published between 2001 and 2021 were included in the search. We combined Boolean operators and wildcard characters appropriately to focus the search and detect plural and singular forms of the same terms in all databases. We also included synonyms or spelling variations. As the index terms varied between databases, the choice of terms was checked both by clinicians with specific expertise in amputation and by reading sentinel articles.

Two reviewers (L.C. and M.M.) separately screened the retrieved records after the completion of the electronic search, starting with titles considered potentially relevant. The reading of the abstracts filtered out the records considered eligible. Papers without abstracts were immediately disqualified as they could not be screened properly. Finally, the full text was screened to find papers relevant to the scope of the review.

A third reviewer (A.P.) helped to resolve inconsistencies in the inclusion and exclusion criteria between L.C. and M.M., and the papers went through the selection process with the full agreement of the authors. In addition, the papers that were finally considered eligible were also discussed with the other author (V.T.) to check their consistency with the aim of this review.

In terms of data extraction, the authors collectively decided what information might be relevant based on the focus of the review, clinical experience, and previously published reviews. Therefore, a table was created to highlight the relevant data. Specifically, L.C. and M.M. separately retrieved the data from each article to complete the table. Then, A.P. and V.T. discussed any inconsistencies. Finally, the authors re-read the full text of each article to ensure the accuracy of the relevant data reported in the tables.

### 2.2. Inclusion and Exclusion Criteria

Articles were considered eligible if they were written in English and published in peer-reviewed journals. Both qualitative and quantitative research was considered, in particular cross-sectional, longitudinal, and intervention studies. More specifically, articles were included in the systematic review if they reported the perspective of adult patients with a lower limb amputation due to a clinical condition (e.g., diabetes or vascular disease). Publications were included if they discussed amputation in relation to psychological aspects (e.g., QoL/HRQoL, anxiety, depression, coping). 

Grey literature and articles dealing with the validation of scales and questionnaires were excluded. Studies involving patients of developmental age (<18 years) and amputations due to traumatic events (car accidents or war) were also excluded. Articles that included upper limb amputation were also excluded from the current systematic review. We also excluded articles that were drug trials or that focused on medical and technical issues. Publications that only considered the carer’s or healthcare professional’s point of view were not included.

## 3. Results

**Figure 1:**
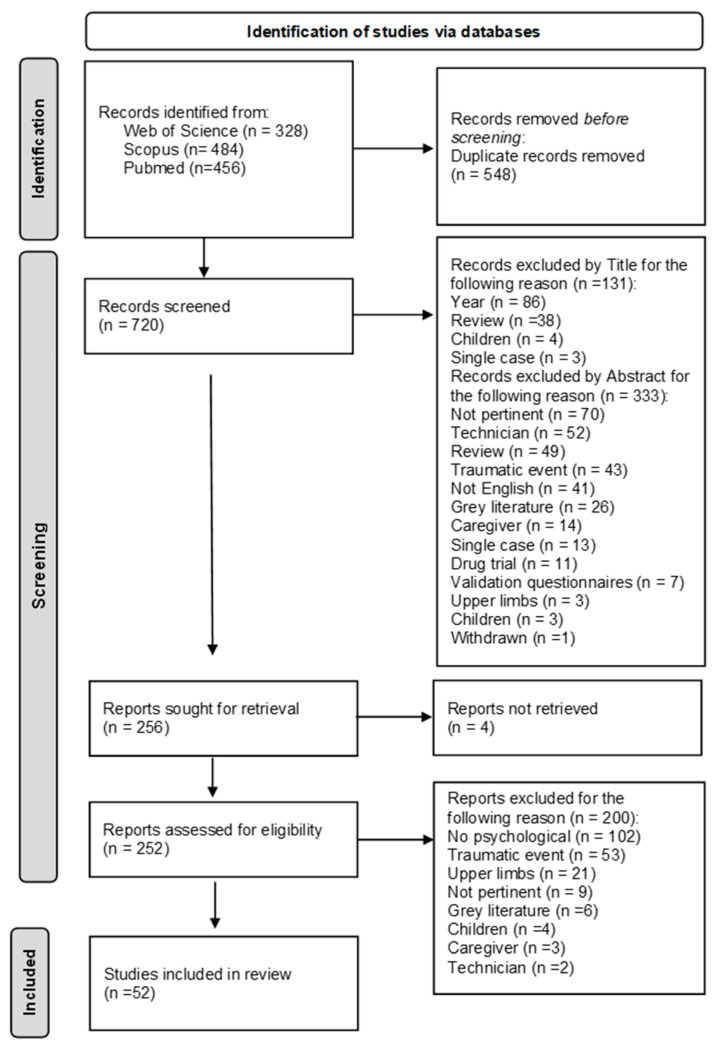
PRISMA flowchart. Represents the systematic review selection process by indicating the number of excluded articles and the reasons.

After searching the databases and removing duplicates, 720 records were found. The titles and abstracts were screened, and 256 eligible articles were found. After full-text screening, 52 publications were eligible for inclusion in this systematic review. The main reasons for exclusion were that many studies did not focus on psychological constructs (n = 102) or reported a traumatic event as the cause of amputation (n = 96) (Figure 1). 

The total number of amputee patients included in this work was 5529, and the sample sizes ranged from studies including 6 to 821 patients (age range: from 36 to 90 years, the majority of whom were aged over 60 years). Not all studies reported whether the sample used prostheses or other walking aids (n° 30, 57.7%). The main cause of amputation was diabetes (n° 28), followed by vascular reasons (n° 19). It should be noted that several articles included samples with different organic causes of amputation.

Table 1 summarises the results in terms of the countries of origin of the studies. In short, most of the studies were conducted in Europe, specifically in the United Kingdom (15.4%) and Portugal (15.4%). More than half of the articles had a quantitative design (75%), the others had a qualitative design (21.2%), and the rest were psychological interventions (3.8%). 

The articles showed that QoL/HRQoL, and psychological adjustment are based on a variety of constructs. It should be noted that since some articles focused on patients’ perceptions of quality of life in general (QoL) and others on quality of life in relation to health status (HRQoL), we decided to keep both acronyms in the results and discussion sections.

**Figure 2:**
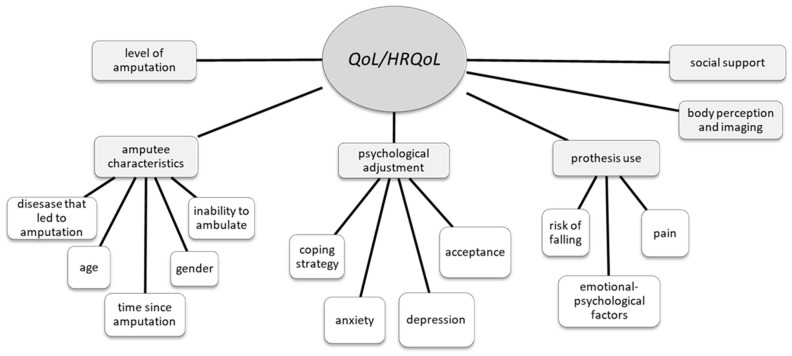
A visual map of factors associated with QoL/HRQoL in LLA for non-traumatic reasons.

Several articles addressed QoL/HRQoL, depression, anxiety, social support, and prosthesis use (Table 2). These topics were reported in both quantitative and qualitative articles. The qualitative articles focused mainly on the subjective experience of being a lower-limb amputee. Focus groups or interviews (either face-to-face or by telephone) were used to explore the constructs of interest. In quantitative studies, there was wide variability in the instruments chosen. The most commonly used instruments were HADS (Hospital Anxiety and Depression Scale) (n° 13), SF-36 (n° 11), and WHOQOL-BREF (World Health Organisation Quality of Life Brief Version) (n° 9). Regarding the functional index, the Barthel scale is the most commonly used and is difficult to relate to a single construct but rather to a broader measure of the patient’s functionality [22,29,30,31,32]. Both quantitative (75%) and qualitative (21.2%) studies highlighted factors linked to QoL/HRQoL. For this reason, we considered QoL/HRQoL to be the core construct of our review (Figure 2).

To provide a clearer understanding, we decided to analyse the results based on the design used in the studies.

### 3.1. Findings from Quantitative Research

In the quantitative articles, a relevant issue is the decrease in QoL/HRQoL soon after the amputation [19,27,42,58,61,64,65]. However, QoL/HRQoL tended to improve over time, especially after 6 months [55,64] or after 1 year [29]. In addition, studies showed a negative association between QoL/HRQoL and anxiety [22,23,26,27,32,44,45,46,48,57] and depression [10,22,23,26,27,32,42,44,45,46,48,57,68]. In particular, studies by Pedras et al. [46] reported that increased preoperative anxiety and postoperative depression were associated with decreased QoL/HRQoL. 

The results showed that factors influencing QoL/HRQoL improvement were social support [11,19,22,23,27,46,47] and prosthesis use [19,27,41,49,55,58,59,60]. The latter was particularly dependent on fear of falling [30,31,55], pain [34], emotional and psychological factors [49], such as coping strategies used [27,49], anxiety [49], and depression [49]. 

Another aspect relevant to QoL/HRQoL in amputees was the level at which the amputation was performed; transtibial amputations correspond to a higher level of health than transfemoral amputations [11,27,63,65,66,67]. Other relevant factors for QoL/HRQoL were: time since amputation [57], persistence of pain [19,27,47,48,57,65], body perception image [27,42,53], coping strategies [22,27,51], age [19,57,58], gender [11,63], inability to walk [57], and acceptance of own illness and clinical conditions [35]. In this context, disease acceptance implied an increase in functional independence and an overall improvement in QoL/HRQoL [35,53]. It has also been described that disease acceptance is lower in subjects who still experience pain [36].

Appendix A (Table A1) shows the quantitative articles included in the current systematic review.

### 3.2. Findings from Qualitative Research

From a qualitative point of view, social support [39,54] and emotional support [33] seem to play an important role. Other findings highlighted the amputation experience in general, from the time when the person made the decision to undergo amputation to the experience of regaining partial independence [50]. During this journey, amputees may experience a loss of control over their lives, both at the level of social relationships and their role in society and at the physical level (i.e., mobility and functionality) [6,54]. The loss of mobility due to amputation can lead to isolation, which in turn threatens future expectations [37,54]. The study by Torbjörnsson et al. [38] showed that some people perceived greater benefits (e.g., reduced pain, reduced risk of death) than the costs of the amputation.

Appendix B (Table A2) shows the qualitative articles included in this systematic review.

### 3.3. Findings from Psychological Intervention Studies

Two intervention studies were included in this systematic review. These looked at the rehabilitation period, which focused mainly on occupational/physiotherapy interventions [56] and desensitisation [40]. The first included a change in QoL/HRQoL and functional independence, and the second led to a reduction in pain levels.

Appendix C (Table A3) shows the psychological intervention studies included in this systematic review.

## 4. Discussion

This systematic review aims to explore QoL/HRQoL and the psychological adjustment of non-traumatic lower limb amputees. Overall, the results led to the identification of several factors related to this health condition. 

From a descriptive point of view, half of the studies were conducted in Anglo-Saxon countries (UK, USA, Canada) and Portugal, suggesting a specific interest in studying QoL/HRQoL and psychological manifestations in people with disabilities or physical limitations. 

In terms of conditions leading to amputation, diabetes and vascular problems were the most common, due to the progressive worsening of clinical conditions associated with these diseases and poor long-term adherence. The majority of articles on LLA focused on psychological adjustment, adherence/compliance and QoL/HRQoL assessment; only two articles investigated educational or psychological interventions. In addition, most studies, both quantitative and qualitative, described QoL/HRQoL as a core construct.

### 4.1. QoL/HRQoL over Time

Overall, QoL/HRQoL was significantly lower in LLA, as reported in previous reviews [69,70]. Nevertheless, results also showed that at some point after amputation (i.e., 6 months to 1 year after surgery), QoL/HRQoL improved [29,55,64]. This improvement has been described as depending on several factors, such as the enhancement of physical performance in terms of functionality and mobility, the emotional and motivational aspects, the social support received, and the amputee’s characteristics. In the literature, there is still disagreement as to which characteristics may play a more relevant role in patients’ psychological adjustment and their QoL/HRQoL: the disease that led to the amputation [26], age [19,57,58], gender [11,63], inability to walk [57], body image [27,42,42,53]. For example, amputation level has been described in previous literature as a key factor influencing QoL/HRQoL. Specifically, higher amputation levels, such as transfemoral, correspond to lower QoL/HRQoL than transtibial amputation [63,65,67,71]. These data may be explained by the fact that transfemoral amputations may require longer periods of rehabilitation to regain function and independence than trans-tibial amputations. 

In this context, another relevant aspect that emerged from this review is the use of prostheses and the ability to walk again. It is well known that prostheses are a means of regaining independence and motor function and therefore have an overall impact on QoL/HRQoL. In this regard, Davie-Smith et al. [69] found that the ability to walk influenced participation in social activities and the ability to live independently. From a daily clinical perspective, prosthesis use depends on comorbid conditions, social functioning, amputation level, and the patient’s motivation. In our review, the factors that negatively influenced prosthesis use were the presence of pain [34], the risk of falling [30,31,55], anxiety [49], and depression [49]. Coping behaviours may play a positive or negative role too, depending on the strategies used [27,49]. These factors were also found by Luza et al. [72], who showed that physical adaptation may also depend on age, education level, and daily use of the prosthesis. It is important to note that not all articles included in our review specified whether amputees used a prosthesis or other walking systems. According to us, this lack of detail is a gap that further research should fill in order to better understand patients’ needs and possible difficulties and resistance towards specific types of devices or interventions proposed to them.

### 4.2. QoL/HRQoL and Psychological Constructs

As shown in Table 2, most studies have investigated and confirmed the role of depression and anxiety in this clinical population. These data are consistent with what is known about amputees, regardless of the reason for surgery. Indeed, in a recent review by Sahu et al. [73], symptoms of anxiety and depression were present and improved over time in lower-limb traumatic amputees. Anxiety and depressive symptoms are negatively correlated with QoL/HRQoL, highlighting how these constructs are risk factors for the success of the rehabilitation process [23]. Thus, mood dysregulation has an impact on motivation and negatively affects adaptation. It should be noted that higher levels of depression have been found in this population than in other hospitalised patients [26]. In addition, Pedras’ studies [22,45,46] have shown that the presence of both increased preoperative anxiety and postoperative depression correlates with a decrease in QoL/HRQoL. Thus, anxiety and depressive symptoms deserve special attention as they may affect rehabilitative outcomes and pose barriers to patients’ motivation and engagement. 

An interesting aspect that emerged from the articles concerns knowledge about the amputation process [13,17]. Amputees in Torbjörnsson’s group [38] reported that they did not feel involved in the amputation decision and had not received enough information, which made the acceptance process difficult. In fact, amputation is a complex and personal experience that involves regaining independence and accepting the loss of a body part [34]. The amputee is therefore required to reconfigure her/his role at work, in the family, and in society [37,54]. Acceptance is thus a multifaceted process of life reorientation with respect to the new condition [6]. Indeed, acceptance of the illness involves coping with difficult experiences that may include grief, anxiety, and embarrassment [74]. The ability to cope with these internal experiences has an impact on maintaining high QoL/HRQoL [35]. For example, amputees who still reported pain were found to have lower disease acceptance [36]. Subjects who received more benefits from the amputation (such as less pain and a reduced risk of death) reported greater satisfaction and acceptance [17,35,38,53]. 

Coping strategies also play a key role in the acceptance process. Indeed, the amputation experience involves a loss of control over one’s life; therefore, the amputee tries to reorganise himself with respect to the new situation by using different coping strategies [6]. Coping strategies appear to contribute to a person’s psychological well-being and reduce the negative impact of amputation [27]. QoL/HRQoL is influenced by problem-focused strategies [27,50] and motivation to regain independence [17]. Therefore, it is recommended to propose and investigate psychoeducational interventions aimed at promoting and fostering appropriate problem-solving and coping skills in dealing with the new challenges of daily living.

Furthermore, body image, which is still poorly investigated in non-traumatic LLA, was found to be a predictive component of QoL/HRQoL [27]. Body image negatively correlates with QoL/HRQoL [27] and has an impact on psychosocial outcomes [43]. It must be said that the few results on body perception and body image suggest the need for further research to pay more attention to the body component in this clinical population. The body without a limb part needs a new tool with which the patient must explore the world and start the process of acceptance. Thus, the patient must find a new psychophysical balance and a satisfactory QoL/HRQoL.

Social support can be considered a protective factor and is associated with better mental health [19]. It mitigates negative outcomes and has been positively correlated with resilience—the ability to cope with and overcome a traumatic event [42]. Social support refers not only to family and friends but also to other amputee patients. In particular, peer support allowed them to compare with each other, create clearer and more realistic expectations about the amputation [13], and help define practical and achievable goals, helping to accept the changed situation [38]. These findings suggest the importance of a global approach to patient care, including attention to the social network and carers who can help the amputee cope with the new health status. Again, findings highlight the importance of a biopsychosocial approach that focuses not only on medical and rehabilitation needs but also on psychological and social needs [75].

Finally, only a few articles reported on interventions in this clinical population. These studies focused on rehabilitation time, with an emphasis on the variation in QoL/HRQoL following multidisciplinary treatments [56] and the reduction of pain through tactile desensitisation [40,76]. Further research is needed to better understand the role of a multidisciplinary approach involving different healthcare professionals (doctor, nurse, physiotherapist, psychologist, and dietician). It is important to understand how rehabilitation interventions could contribute to positive psychological adjustment, taking into account the relevant process of constant self-redefinition throughout life [77].

### 4.3. Future Research, Strengths, and Limitations

Overall, this systematic review is one of the few attempts to synthesise the evidence on various psychological aspects of non-traumatic lower limb amputation. To our knowledge, it is the only review that considers the relationship between QoL/HRQoL and psychological adjustment from a multifaceted perspective, drawing on evidence and suggestions from quantitative, qualitative, and interventional studies. The findings may pave the way for future research and interventions tailored to this clinical population. For example, multidisciplinary programmes are more than welcome in order to promote greater awareness among patients of what this type of surgical intervention will mean for them from a functional, psychological, and social point of view. Making patients more informed, motivated, and strategic can indeed improve adherence to medical and behavioural treatments and thereby improve outcomes, as suggested by the Three Factor Model [78]. These results can be achieved through group classes that provide patients with knowledge on medical, functional, and nutritional aspects, as well as psychoeducation tips to promote the best possible QoL/HRQoL and counteract depression or anxiety. Future research is therefore recommended to test which combinations of interventions may be more effective in maximising the well-being of these patients.

Despite possible merits, there are some limits that need to be discussed. Firstly, there was no quality assessment of the articles, which may lead to the inclusion of poor-quality research or studies with different characteristics. For example, it should be noted that not all articles specified whether participants used prostheses or other walking aids, which may have influenced the relevance given to some factors. This choice was made with the aim of collecting all data on the topic in order to provide suggestions and tips for clinical practise and to suggest well-structured observational and interventional studies. Secondly, no meta-analysis has been performed. Further research is recommended to fill this gap. Thirdly, the comparison of research studies may be challenging due to the differences between healthcare systems and cultures around the world regarding lower limb amputees and rehabilitation interventions. Thus, the results may be biased by the lack of certain cultural considerations that should have explained the decision to investigate only some aspects and neglect others. Fourthly, the choice of search terms may have introduced a further bias, as may the lack of comparison between traumatic and non-traumatic amputations. Finally, there were no differences in the clinical conditions leading to amputation, which may be another aspect to investigate in future research. 

## 5. Conclusions

This review shows that amputation is a complex process, not just a physical event. QoL/HRQoL in lower limb amputees is initially lower but can be improved through various factors: the level of amputation and the medical condition that caused it; general clinical conditions; perceived social support; motivation; individual and social characteristics; and the presence of depression, anxiety, and coping strategies. In addition, QoL/HRQoL may be influenced by the whole rehabilitation process, which in turn may influence amputees’ QoL/HRQoL. The findings highlight the need to develop multidisciplinary interventions that address not only the physical aspect but also the psychological and social dimensions to improve QoL/HRQoL. Future studies could examine changes identified in this review at different stages of the amputation process, particularly from the decision to amputate to physical rehabilitation and then to prosthesis fitting. Overall, according to the authors, these findings provide useful suggestions not only for research but also clinicians: positive psychological adjustment to the daily challenges can promote better adherence, QoL/HRQoL and medical outcomes in amputees.

## Figures and Tables

**Table 1:** Results related to nations.

Nation	HD (Ranking)	n (%) 52
UK	0.932 (13)	8 (15.4%)
Portugal	0.864 (38)	8 (15.4%)
Canada	0.929 (16)	6 (11.5%)
USA	0.926 (17)	4 (7.7%)
Sweden	0.945 (7)	3 (5.8%)
Australia	0.944 (8)	2 (3.8%)
Ireland	0.955 (2)	2 (3.8%)
Netherlands	0.944 (8)	2 (3.8%)
Turkey	0.820 (54)	2 (3.8%)
Poland	0.880 (35)	2 (3.8%)
Serbia	0.806 (64)	1 (1.9%)
Europe	-	1 (1.9%)
France	0.901 (26)	1 (1.9%)
Germany	0.947 (6)	1 (1.9%)
Hungary	0.854 (40)	1 (1.9%)
Jamaica	0.734 (101)	1 (1.9%)
Malaysia	0.810 (62)	1 (1.9%)
Pakistan	0.557 (154)	1 (1.9%)
Romania	0.828 (49)	1 (1.9%)
Saudi Arabia	0.854 (40)	1 (1.9%)
Sudan	0.510 (170)	1 (1.9%)
Taiwan (China)	0.761 (85)	1 (1.9%)
Trinidad and Tobago	0.796 (67)	1 (1.9%)

The HDI (Human Development Index) is based on three dimensions: (a) life expectance at birth; (b) expected years of schooling and mean years of schooling; and (c) gross national income per capita (United Nations Development Programme, http://hdr.undp.org/en, accessed on 20 July 2022).

**Table 2:** Main constructs, in alphabetical order, emerging from the review and related references.

Constructs	Definitions	References
Acceptance	The subject’s awareness of the new physical condition	[1,6,10,13,17,33,34,35,36,37,38,39]
Anxiety	Psychological state characterised by worry and apprehension	[22 ^d^] [23, ^d^] [26, ^d^] [27] [32, ^d^] [36, ^d^] [40, ^d^] [41, ^d^] [42, ^d^] [43, ^d^] [44, ^d^] [45, ^d^] [46, ^d^] [47, ^d^] [48]
Coping strategies	Behaviours used to manage, minimise, and control stressful or negative events	[13,17,22,23,27,37,39,47,49,50,51,52]
Depression	Psychological state characterised by a sad, empty, or irritable mood; may be accompanied by cognitive, behavioural, or physiological changes that affect the person’s daily living	[10, ^b^] [22, ^d^] [23, ^a,d^] [26, ^d^] [27] [30, ^b^] [31, ^b^] [32, ^d^] [36, ^d^] [40, ^d^] [41, ^d^] [42, ^d^] [43, ^d^] [44, ^d^] [45, ^d^] [46, ^d^] [47, ^d^] [48, ^a^] [52] [53, ^a^]
Experience of being a lower-limb amputee	Direct and personal knowledge regarding being an amputee	[1,6,17,33,34,37,38,39,50,54]
Prosthesis use	Use of any type of prosthesis by amputees	[11, ^f^] [13,17] [22, ^f^] [27, ^f^] [29,30,31,34,36,37,38,41,49,53,54,55,56] [57, ^f^] [58,59,60]
QoL/HRQoL	Level of perceived well-being in relation to the socio-cultural context in which individuals live. HRQoL specifically focuses on health aspects	[11, ^e,g^] [19, ^e,g^] [22, ^e^] [27] [29, ^e^] [35, ^g^] [36, ^g^] [42, ^g^] [43, ^g^] [44, ^e^] [46] [47, ^g^] [55, ^e^] [56, ^e^] [57, ^e^] [58, ^c^] [59, ^c^] [60, ^e^] [61] [62, ^e^] [63, ^g^] [64, 65] [66, ^e^] [67, ^c^] [68, ^g^]
Social support	Perceived support received from family and friends	[1,11,17,19,22,23,27,29,30,34,37,39,46,52,54,68]

Note. The more frequent questionnaires used in the review were signed in the table: Beck Depression Inventory (BDI) ^a^, Centre for Epidemiologic Studies Depression Scale (CES-D) ^b^, European Quality of Life 5 Dimensions 3 Level Version (EQ-5D-3L) ^c^, Hospital Anxiety and Depression Scale (HADS) ^d^, Short Form Health Survey 36 (SF-36) ^e^, Trinity Amputation and Prosthesis Experiences Scales (TAPES) ^f^, World Health Organisation Quality of Life Brief Version (WHOQOL-BREF) ^g^.

## Data Availability

The data that support the findings of this study are available from the corresponding author, M.M., upon reasonable request.

## Appendix A

**Table A1:** The quantitative articles included in the systematic review.

Author(s), Year [References]	Country of the Study	Subjects	Psychological Construct Assessed	Study Design	Main Results
Abdelgadir et al., 2009 [61]	Sudan	Recruited from outpatients diabetic clinic, 60 (40 m, 20 f, 57.4 age) with LLA, 60 (23 m, 37 f; 52.8 age) reference.	QoL/HRQoL (HRQoL assessment; SOC-13; the symptom check-list), diabetic.	Quantitative (cross-selection study).	The QoL/HRQoL of the diabetic subjects with LLA was found to be low compared to the diabetic reference subjects.
Barnett et al., 2013 [55]	UK	Seven male (56.1 ± 14.9 age) unilateral transtibial amputees that had followed a course of rehabilitation.	QoL/HRQoL (SF-36), prothesis (PEQ), and self-perceived ability without falling (mFES).	Quantitative (longitudinal).	Observable and clinically meaningful changes in QoL/HRQoL and fall efficacy were reported, resulting in large effect sizes. Mental health improved relative to physical health, suggesting that increases in physical health over time would be required to produce further increases in overall QoL/HRQoL. Changes in the indices of fall effectiveness were equally associated with physical and mental health.
Boutoille et al., 2008 [62]	France	Twenty-five (68 age) patients hospitalised as amputees; 9 (70 years mean age) outpatients with diabetic foot ulcers.	QoL/HRQoL (MOS-SF-36).	Quantitative (cross-selection).	The impact of amputation on QoL/HRQoL is no worse than if the patient had a chronic foot ulcer. The pain in the ulcer group is probably mainly due to peripheral arterial disease.
Callaghan et al., 2008 [49]	UK	Recruitment 166 (69.3% m, 30.7% f; 66.73 age), 1 month follow-up post-discharge 143 (69.2% m, 30,8% f, 66.47 age), 6 months follow-up 120 (69.2% m, 30.8% f; 66.39 age).	Psychological variables within the CS-SRM (IPQ-R) prosthetic use (FMA).	Quantitative (longitudinal predictive design).	Improvements in all prosthesis use outcome variables were observed between 1 and 6 months of follow-up. The CS-SRM causal attribution cognitive representations (i.e., risk factors and emotional-psychological factors) were influential in determining prosthetic use at 6 months but not at 1 month post-discharge. There was a significant relationship between risk factors and prosthesis use, in contrast to a significant relationship between emotional-psychological factors and prosthesis use. Although activity levels with a prosthesis steadily improved between the two follow-up assessments, they had not significantly returned to preoperative activity levels.
Couture et al., 2012 [53]	Canada	Twenty-one individuals with a unilateral LLA due to vascular disease were evaluated during the first 2 weeks of their hospitalisation (T1), 2 weeks before discharge from rehabilitation (T2) (19), and 2–3 months post-discharge from rehabilitation (T3) (16).	Functional independence (SMFA; ADL; IADL); locomotor capabilities with prosthesis (Locomotor Capabilities Index), depression (BDI), and body image (BIQ).	Mixed-method (longitudinal design).	Many people (60%) who have had a LLA consider this event positive and are able to identify benefits associated with the amputation. Qualitative and quantitative findings suggest that the appraisal of the amputation is maintained over time. Individuals who rated their amputation as positive had better functional independence (T1) and greater body image satisfaction (T3).
Cox et al., 2011 [63]	Jamaica	Eighty-seven (35 m, 52 f), Age ranged between 43–86. Sixty-four had below-knee amputations, and 23 had above-knee amputations.	QoL/HRQoL (WHOQOL-BREF), functional independence (FIM).	Quantitative (cross-selection study).	Below-knee amputees were found to have significantly higher levels of functional independence and QoL/HRQoL measures than above-knee amputees. Female amputees had surgery at an earlier age than males and have coped better with LLA.
Cruz et al., 2021 [19]	Portugal	One hundred and six (78 m, 28 f, 72 age).	QoL/HRQoL (WHOQL-BREF, SF-36).	Quantitative (cross-selection study).	Patients had an overall post-amputation QoL score of 79.0 out of 100, which was correlated with their own subjective assessment of QoL/HRQoL. The results suggest that improvements in physical health and functioning are positively correlated with social and psychological health, and that better social support is associated with higher post-amputation psychological health scores.
Deans et al., 2008 [11]	UK	Twenty-five (20 m, 5 f) participants recruited with a letter invitation	Physical activity (TAPES, SF-36), QoL/HRQoL (WHOQOL-BREF).	Quantitative (cross-sectional descriptive study).	Weaker-than-expected association between physical activity and quality of life in LLA. This study supports increasing physical activity in this patient group, as long as social interaction is not compromised.
Dillon et al., 2019 [57]	USA	One hundred and twenty-three (84 m, 39 f, 55.3 age); 42 partial foot amputations; 81 transtibial amputations.	Experience LLA (TAPES), QoL/HRQoL (SF-36v2, PROMIS-29v2).	Quantitative (cross-sectional survey).	Variation in QoL/HRQoL was associated with time since amputation, fatigue, anxiety, depression, pain interference, and physical function in people with PFA or TTA.
Fortington et al., 2013 [64]	Netherlands	Vascular surgeons from hospitals were asked to refer people who were undergoing amputation. Eighty-two patients (55 m, 27 f, 67.8 age); 6 months, 46 patients; and 18 months, 35 patients.	QoL/HRQoL (RAND-36).	Quantitative (multicentre, longitudinal study).	The results show that significant improvements in QoL/HRQoL can be achieved after LLA. QoL/HRQoL scores reflect the difficult situation faced at the time of amputation, with only mental health and general health scoring above 50 (out of 100). However, for those who survive, there are significant improvements across the different domains, with most of the change occurring in the first 6 months.
Juzwiszyn et al., 2021 [35]	Poland	Ninety-nine patients (23 m, 76 f, 72.1 age) had all undergone diabetes-related LLA.	QoL/HRQoL (WHOQOL-BREF), nutritional (MNA), and acceptance of illness (AIS).	Quantitative (cross-sectional study).	The better the quality of life in all domains, the better the acceptance of illness. The less malnourished the patient, the better the QoL in all domains.
Kizilkurt et al., 2020 [27]	Turkey	Sixty-five patients who had undergone amputation because of an infected diabetic foot ulcer.	QoL/HRQoL (SF-36), prosthesis (TAPES), coping (COPE), social support (MSPSS), self-assessment (RSES, ABIS), and anxiety-depression (PHQ-SADS).	Quantitative (cross-sectional study).	The results show that physical and mental QoL/HRQoL were reduced after LLA compared to the normal population. The presence of phantom limb pain, additional medical conditions, and level of prosthesis were found to be factors associated with QoL/HRQoL. Depression and anxiety scores, body image, self-esteem, perceived social support, problem-focused and dysfunctional coping strategies, post-prosthetic activity limitation, and prosthetic satisfaction were found to be related to QoL/HRQoL.
Knežević et al., 2015 [65]	Serbia	Fifty-six subjects (aged 30 to 83). The experimental group consisted of 28 (21 m, 7 f) patients, their average being unilateral amputation of the lower extremities, while the control group consisted of 28 people with intact lower extremities.	QoL/HRQoL (RAND).	Quantitative (cross-sectional study).	The QoL/HRQoL of patients with LLA is significantly reduced compared to the control group, despite a fairly successful and satisfactory restoration of walking function and relative independence in daily activities. There is no significant difference between genders in overall physical and mental function, whereas patients with different levels of amputation differ in physical function and general health. Patients with transtibial amputations are more functional and have better general health than patients with transfemoral amputations.
Krzemińska et al., 2021 [36]	Poland	One hundred and seventeen patients were recruited, but those who could not continue their participation. The group completed 100 patients (64 m, 36 f).	QoL/HRQoL (WHOQOL-BREF), acceptance of illness (AIS), anxiety-depression (HADS), and pain (VAS 10 cm).	Quantitative.	Pain and its intensity are associated with QoL/HRQoL in patients with complicated diabetic foot syndrome. More severe pain was associated with lower QoL/HRQoL in the physical and psychological domains 6 months after amputation and with lower QoL/HRQoL in the environmental domain 12 months after amputation. Disease acceptance was lower in patients with more severe pain at all stages of the study. Pain intensity was associated with more severe affective disorders at the 6 month follow-up.
Larner et al., 2003 [41]	UK	Forty-three (66.35 age) successive LLA suffering from peripheral vascular disease with or without diabetes were admitted to a multidisciplinary rehabilitation programme. Thirty-one learned to use a prosthesis (prosthetic group, 22 m, 9 f), 12 did not (non-prosthetic group).	Anxiety-depression (HADS), physical rehabilitation (RLOC), and learning ability (KOLT).	Quantitative (one-sample design).	The study showed that the combination of amputation level (transfemoral or transtibial) and poor learning ability had a predictive rate of 81% for mobility after rehabilitation in unselected cases, including those who were medically unfit. None of the psychological measures other than the KOLT were predictive of the ability to learn to use a limb.
Madsen et al., 2018 [29]	Sweden	At the baseline, there were 59 participants. Three months follow-up 41, 6 months follow-up 41, and 12 months follow-up 38 (28 m, 10 f, 67.8 age).	QoL/HRQoL (SF-36, GSE), daily activity (Barthel Index).	Quantitative (prospective cohort study design).	Patients have better QoL/HRQoL 12 months after amputation compared to 1 month before amputation in all domains except physical function and are significantly more dependent on assistance with activities of daily living measured at the group level. Patients were more dependent on assistance in four activities at all three time points measured and had significantly worse function in toileting, self-bathing, walking, and transferring from bed to chair at 12 months.
Makai et al., 2019 [23]	Hungary	Twenty-nine participants (20 m, 9 f, 51 age) had LLA due to diabetes mellitus.	Depression (BDI), anxiety (HADS), resilience (CD-RISC), social support (MOS-SSS), coherence (SOC), and positive-negative affect (PANAS).	Quantitative (follow-up study).	The study takes a novel resilience-based approach to the protective and risk factors that influence outcomes. Depression and anxiety were found to be risk factors at both follow-up measures, while protective factors such as positive affectivity, social support, and a sense of coherence were positively associated with resilience, indicating their important role in successful adjustment. The importance of these protective factors for resilience was found to increase 6 months after the intervention.
McDonald et al., 2014 [42]	Australia	Participants were recruited predominantly through a diabetes or amputee member’s association. The group with diabetes and amputation was 50 (78% m, 63 age); the group with diabetes and without amputation was 240 (68% m, 64.65 age).	Depression-anxiety (HADS), QoL/HRQoL (WHOQOL-BREF), body image (BIDQ).	Quantitative (multivariate design).	The psychosocial distress of people with an amputation is more pronounced than that of people with diabetes who have not had an amputation. The presence of an objective change to the body has an impact on an individual’s body image, but the overall poorer health of people with an amputation may be a better explanation for greater depression and poorer physical quality of life than the amputation itself.
McDonald et al., 2021 [43]	Australia	Study participants were sent an invitation by a diabetes member’s organisation. Two hundred and twelve responded (60% m, 64.4 age). Individuals with amputation who were patients of a local hospital, a prosthetics clinic, and members’ organisations were invited to participate; 227 responded (70% m, 58.54 age).	Depression-anxiety (HADS), QoL/HRQoL (WHOQOL-BREF), body image (BIDQ, BIQ), physical appearance (ASI-R).	Quantitative (cross-selection).	Body image disturbance, personal investment, and self-ideal discrepancy all independently and directly predicted psychosocial outcomes, over and above demographic and medical factors.
Miller et al., 2001 [30]	Canada	Four hundred and thirty-five (309 m, 126 f, 62 age).	Social support (ISEL), daily activity (Barthel Index), depression (CES-D), confidence (ABC scale), mobility (PEQ-MS, Houghton Scale), and social activity (FAI).	Quantitative (population-based survey and chart review).	Balance confidence is important for mobility and social activity. Balance confidence was found to be more important than fear of falling. Reduced balance confidence was associated with reduced participation in social activities.
Miller et al., 2001 [31]	Canada	Four hundred and thirty-five (71% m, 62 age); of these, 228 fell in the previous 12 months; 207 had no fall.	Daily activity (Barthel Index), depression (CES-D), and fall.	Quantitative (cross-selection study).	People who had been amputated more recently had a higher risk of falling than those who had had their prosthesis for longer. Joint and back pain was associated with a 1.67 to 1.96 times higher risk of falling.
Nazri et al., 2019 [66]	Malaysia	Ninety-four (52 m, 42 f, age ranged from 38 to 85) patients with diabetes were admitted to the orthopaedic wards and planned for amputation. Thirty-six major amputations and 58 minor amputations.	QoL/HRQoL (SF-36).	Quantitative (cross-sectional study).	The walking ability and dependence of patients with diabetes after minor amputation were better than after major amputation at 6 months. Minor amputees had more pain and poorer social function than major amputees. The QoL/HRQoL of minor amputees was better than that of major amputees in the domains of physical functioning, general health, emotional health, and mental health.
Pedras et al., 2018 [32]	Portugal	At baseline (T0), 179 (127 m, 52 f, 66.4 age) patients with diabetic foot ulcers participated; of these, 76 had already been amputated (23.7% and 76.3% major and minor LLAs, respectively). One hundred and thirteen patients participated at T1, approximately one month after surgery.	Depression-anxiety (HADS).	Quantitative (longitudinal study).	Prior to surgery, patients had higher levels of anxiety than depression. In terms of anxiety symptoms, they decreased from T0 (63.7%) to T1 (41.6%). Despite clinical and demographic variables, preoperative anxiety and depression were found to be predictors of postoperative anxiety and depression.
Pedras et al., 2018 [22]	Portugal	Two hundred and two (72.3% m, 66.2 age); 57.9% had already been amputated in the past.	Depression-anxiety (HADS), daily activity (Barthel Index), and QOL/HRQoL (SF-36, index: PCS, MCS).	Quantitative (cross-sectional study).	The results showed an association between anxiety symptoms, depression symptoms, functional level, and MCS and PCS. The results showed the simultaneous influence of socio-demographic, clinical, and psychological variables on MCS and PCS, in line with the biopsychosocial model.
Pedras et al., 2020 [45]	Portugal	One hundred and forty-nine (t0) patients proposed for amputation surgery were identified (105 m, 44 f, 65.5 age). T1 144 (102 m, 42 f, 65.6 age); T2 107 (74 m, 33 f, 64.7 age); and T3 96 (71 m, 25 f, 63.7 age).	Depression-anxiety (HADS).	Quantitative (longitudinal and multicenter studies).	Neither anxiety nor depressive symptoms were significant predictors of re-amputation; the period in which emotional symptoms had the greatest impact on clinical outcomes was pre-surgery. Preoperative anxiety levels had a greater impact on healing than postoperative anxiety levels. The results showed that the 10 month mortality rate was 9.4%, the re-amputation rate was 27.5%, and the healing rate was 61.7%.
Pedras et al., 2019 [46]	Portugal	Two hundred and six patients were evaluated 24 h (median: 1 day) before surgery (t0), 1 month after surgery (t1), 6 months (t2), and 10 months (t3) after surgery during their follow-up consultations at the hospital. Only 86 (63 m, 23 f, 63 age) patients completed four assessments.	Traumatic stress (IES-R), depression-anxiety (HADS), daily activity (Barthel Index), social support (SSSS), and QOL/HRQoL (SF-36, index: PCS, MCS).	Quantitative (multicenter, longitudinal study).	The results revealed that increased anxiety before surgery and symptoms of depression 1 month after surgery were associated with lower MCS 10 months after surgery. Functional level before and 1 month after surgery, traumatic stress symptoms after surgery, and satisfaction with social support 6 months after surgery were associated with PCS 10 months after surgery. Social support was a mediator between traumatic stress symptoms and PCS.
Pedras et al., 2018 [44]	Portugal	The sample comprised 86 (63 m, 23 f, 63 age) patients that consecutively participated in all assessments from t0 to t3.	Activity of daily (Bathel index), depression-anxiety (HADS), traumatic stress (IES-R), social support (SSSS), coping (WOC), and experience of amputation (TAPES).	Quantitative (longitudinal, multisite study).	Psychological assessment and intervention are recommended, especially in the preoperative period, to control the negative association between anxiety symptoms and social adjustment 10 months after LLA. The results indicated that traumatic stress symptoms 1 month after surgery were negatively associated with functional and social adjustment, i.e., overall psychosocial adjustment to LLA 10 months after surgery.
Pedras et al., 2014 [76]	Portugal	Two hundred and six patients (149 m, 57 f, 66 age) were hospitalised due to a diabetic foot ulcer and were referred for amputation surgery.	Pain (BPI, DN4).	Quantitative (cross-sectional study).	The maximum pain intensity reported by patients was significant, and overall, it was found that patients lived with pain on a daily basis. The oldest patients with a longer duration of ulceration reported higher pain intensity. Patients with more hospitalisations and with a neuroischemic foot reported higher pain too.
Pickwell et al., 2016 [67]	Europe	Eight hundred and twenty-one patients were included, of whom 145 had minor amputation (64.5 age) and 676 had conservative treatment.	QoL/HRQoL (EQ-5D-3L).	Quantitative (multicenter, prospective study).	Nonhealing was associated with no change in QoL/HRQoL as measured by the EQ-5D, whereas ulcer healing was associated with improvement in QoL/HRQoL. The study found that minor amputations were not associated with a negative impact on QoL/HRQoL.
Pran et al., 2021 [58]	Caribbean countries	One hundred and thirty-four (62% m, 38% f, 63 age) individuals with LLA.	QoL/HRQoL (EQ-5D-3L).	Quantitative (cross-sectional study).	The QoL/HRQoL after LLA and independent mobilisation with a prosthesis were problematic in this population. Factors negatively associated with QoL/HRQoL after LLA include increasing age and non-ambulatory patients. Ambulation with a prosthesis was found to be associated with better QoL/HRQoL in both transtibial and transfemoral patients.
Richardson et al., 2007 [51]	UK	Sevety-seven patients were referred, and 52 amputees completed the 6 month interview. Forty-one patients (78.8%) reported phantom limb pain (PLP) at 6 months (63.3% m, 63.8 age).	Pain (MPQ), coping (CSQ).	Quantitative (prospective study).	Preamputation physical and psychological factors have been found to be associated with the development of PLP after LLA. Preamputation pain may play a role in the development of PLP, but the relationship between its intensity and duration needs to be further elucidated. Passive coping styles have previously been found to be prevalent in PLP patients. This study suggests that these were present prior to amputation and may have influenced the development and maintenance of PLP.
Schrier et al., 2019 [47]	Netherlands	Thirty-one adult patients (6 m, 25 f, 37.5 age) with longstanding, therapy-resistant complex regional pain syndrome type-I (CRPS-I) underwent an amputation.	QoL/HRQoL (WHOQOL-BREF), resilience (CD-RISC), depression-anxiety (HADS), and psychological distress (SCL-90-R).	Quantitative (cross-selection study).	Poor amputation outcomes in long-standing treatment-resistant CPRS-1 are associated with psychological factors. These factors are not specific to CRPS-I recovery or rehabilitation. Major life events are not associated with poor outcomes, although half of the participants had experienced major life events.
Senra 2012 [10]	Portugal	Forty-two (35 m, 7 f, 61 age) adult patients, followed up at the rehabilitation medicine service of a general public hospital.	Depression (CES-D), experienced LLA and its implications for their self-identity (two face-to-face interviews).	Mixed method (qualitative: thematic and categorical analysis proposed by Bardin).	A significant association was found between the main variables related to the amputation experience and depressive levels. Higher levels of depression were found in patients who reported greater self-awareness of the impairment, lower self-identification with the impairment, inadequate social support, and poor well-being.
Singh et al., 2007 [26]	UK	A cohort of 105 (72 m, 33 f, 62.9 age) consecutive admissions to an amputee rehabilitation.	Depression-anxiety (HADS).	Quantitative (cohort study).	The initial prevalence of depressive symptoms was 27%, more than three times the general hospital admission rate, which ranged from 3.6% to 10.6%. Isolation was associated with anxiety and other medical conditions associated with depression but not with amputation level, success of limb fitting, age, or gender.
Sucalã et al., 2010 [48]	Romania	The initial study sample included 132 participants (71% m, 62.7 age).	Depression (BDI-II), anxiety (STAI-Form Y), and pain (MPQ).	Quantitative.	The results show that amputees experience high levels of preoperative distress, with a large proportion of participants scoring in the clinical range on measures of depression and anxiety. The high levels of preoperative distress decrease significantly after surgery, although they remain in the clinical range.
Torbjörnsson et al., 2020 [59]	Sweden	Ninety-eight patients were included in the study, but of the 73 (44 m, 29 f) who completed the follow-up, 56 had a prosthesis, and 53 patients used it at follow-up.	QoL/HRQoL (EQ-5D-3L), prosthesis use (Stanmore Harold Wood mobility grade; Houghton scale).	Quantitative (longitudinal).	Patients who were able to walk or use their prosthesis or walking aids (e.g., wheelchair) to move independently had improved QoL/HRQoL one year after amputation.
Vincent et al., 2010 [52]	Canada	Ten (8 m, 2 f 71 age) ambulatory and non-ambulatory participants using different assistive devices to move.	Support (ISEL), social support (MOS Social Support Survey), coping (WCQ), pain (BPI), depression (Yesavage Geriatric Depression Scale), hand-grip strength (dynamometer), sensitivity of the intact foot (Semmes–Weinstein Monofilament Test), performing activities (LCI), time to get up (TUG), balance (BBT), mobility (AMP), effective mobility (LIFE-H, LSA, HPA).	Quantitative (observational and transversal design).	The results show that it was only in groups with moderate and low effective mobility that three or more of a specific subset of modulators were observed: living alone, no prosthetic rehabilitation, low social support, no coping strategy for social support, general pain, low strength in one arm, and low sensitivity in the remaining limb.
Wukich et al., 2017 [60]	USA	Eighty-one patients, of whom 41 (28 m, 13 f 53 age) completed preoperative and postoperative outcome, 40 (32 m, 8 f, 54.5 age). There were no significant differences between the two groups. Twenty of 81 patients (24.7%) died during the median follow-up period of 145.3 weeks.	QoL/HRQoL (SF-36), ability (FAAM).	Quantitative.	Patients with diabetic foot complications had a lower self-reported quality of life than patients with diabetes without foot complications. Those who walked with a prosthesis were six times more likely to improve their SF-36 PCS score and 14 times more likely to improve their overall FAAM score than patients who did not walk.
Zaheer et al., 2020 [68]	Pakistan	Seventy amputees were recruited (53 m, 17 f, 37.9 age).	QoL/HRQoL (WHOQOL-BREF, PHQ-9).	Quantitative (cross-sectional).	The participating amputees experienced significant life changes that negatively affected all areas of their QoL/HRQoL. Participants suffered from mild to moderate depression. The amputees’ QoL/HRQoL and depression scores were negatively correlated (*p* < 0.05), i.e., those with high depression scores had low QoL/HRQoL scores and vice versa.

## Appendix B

**Table A2:** The qualitative articles included in the systematic review.

Author(s), Year	Country of the Study	Subjects	Psychological Construct Assessed	Study Design	Main Results
Abouammoh et al., 2021 [1]	Saudi Arabia	Fourteen (9 above knee, 3 below knee, and 1 at ankle level), between 26 and 71 years.	Experiences, needs, social, and psychological adjustment; physical and psychological support (interviews).	Qualitative (phenomenological).	Patients needed a balanced environment for the healthy expression of their emotions. Moreover, their physical and emotional symptoms could be alleviated by cultural and spiritual traditions. Depressive reactions could be minimised through patient education.
Canbolat et al., 2021 [6]	Turkey	Twelve (9 m, 3 f; 61.3 age).	Experience of LLA (semi-structured interview).	Qualitive (phenomenological design).	Three key themes emerged from this study that encapsulate the lived experience of people with LLA: loss of control over one’s life, dreams versus reality, and expectations for the future.
Columbo et al., 2018 [50]	USA	Twenty (17 m, 3 f, 65 age). Most underwent below-knee amputations.	Experience of LLA (semi-structured interview).	Qualitative (mixed-methods approach: structured interview with 20 patients to examine areas and then a focus group).	Participants described the amputation experience as beginning before surgery with the decision to amputate and ending when they had regained what they perceived to be functional independence. These findings suggest that the recovery process extends beyond the period of inpatient physical rehabilitation and identify potential areas for specific recovery and functional outcome improvement.
Delea et al., 2015 [33]	Ireland	Ten participants were recruited from the prosthetic, orthotic, and limb absence rehabilitation programmes. (male, 58 age).	Experiences of people with diabetes and LLA (semi-structured interview).	Qualitative (inductive thematic analysis).	Most of the participants expressed a need for emotional support in addition to the medical management of their condition. There was considerable variation in the provision of foot care services and supplies from region to region, reflecting the current models of service provision in Ireland.
Gallagher et al., 2001 [34]	Ireland	Fourteen (6 m, 8 f), age between 20 and 50). Five participants had an above-knee prostheses, seven had below-knee prostheses, and two had bilateral amputations. All participants had had their prosthesis for more than 5 years.	Experience LLA and prosthesis (focus group).	Qualitative.	A related theme that emerged was the importance of a reliable prosthetic limb. The information from the focus groups emphasises that, in addition to financial and practical concerns, the emotional and psychological impact of amputation is of paramount importance. It can be seen that they mourn the loss of a visible body part and the loss of function, as well as the impact of amputation on lifestyle and body image. Even if the prosthesis has been a constant and useful feature, it never truly replaces the limb.
Liu et al., 2010 [37]	Taiwan	Twenty-two (age 70.6) Most of the participants were men (68.2%). At the follow-up interviews, there were 19 participants.	Experience LLA (interviews were conducted face-to-face in a prosthetic rehabilitation centre 4–8 weeks postoperation).	Qualitative (phenomenological)	High levels of uncertainty and low levels of knowledge and perceived control contributed significantly to participants’ increased psychological distress, even after successful surgery. The study identified emotional and physical challenges at different stages of psychological adjustment, including sadness, depression, anxiety, anger, frustration, helplessness, increased pain, changes in appetite and sleep problems, and the experience of suffering. Suffering appeared to be related to threats to the future, perceived personal integrity, and a sense of wholeness.
MacKay et al., 2020 [54]	Canada	Thirty-five individuals with dysvascular LLA (23 m, 12 f, 62 age).	Experiences dysvascular LLA (semi-structured interview).	Qualitative (content-analysis approach).	Social support, accessibility, and socio-economic factors played a role in people’s experiences, suggesting that there are opportunities to optimise these factors to improve people’s lives and mitigate negative outcomes. Participants discuss isolation in terms of reduced mobility and independence (not being able to drive), barriers in the built environment, and changes in their social relationships and roles (no longer working).
Ostler et al., 2013 [13]	UK	The 8 participants were inpatients (6 m, 2 f, 51 age).	Expectations of the rehabilitation process (semi-structured interviews).	Qualitative (thematic analysis).	Patients’ expectations after LLA appear to be vague and uninformed, which can lead to uncertainty and passivity. It appears that patients’ expectations are formed through contact with other amputees and health professionals or through information generated by us. High expectations may be an important part of psychosocial coping after amputation, and full management of expectations may be a longer-term process.
Radenovic et al., 2021 [17]	Canada	Nine individuals with LLA (7 m, 2 f, 59 age).	Experience LLA (interviews: 2 of 9 were conducted in person).	Qualitative (descriptive and discovery-oriented approach).	Participants highlighted the crucial role that inpatient rehabilitation can play in preparing them for life in the community by developing the basic skills, peer relationships, and education needed to succeed at home. Factors that participants felt had either helped or hindered their initial experiences in the community included their physical abilities, coping strategies, social support, and access to resources.
Torbjörnsson et al., 2016 [38]	Sweden	Thirteen patients (9 m, 4 f, 73 age).	Experience of an amputation due to peripheral arterial disease (PAD)(interviews: eight were conducted in hospital and the rest at the participant’s home).	Qualitative (content analysis).	This study shows that patients who underwent LLA for PAD experienced a severe lack of knowledge about the process after the amputation, about the procedure, its benefits, possible complications, and what to expect from life after amputation. Most participants were happy with their decision to have an amputation, and some even said they wished they had done it earlier.
Washington et al., 2014 [39]	UK	Four male (64.8 age) and 2 female (69 age).	Experiences of people with LLA, diabetes, and/or peripheral vascular.	Qualitative (phenomenology).	In addition to the impact of the amputation, participants reported difficulties with their underlying health conditions, and the transition from hospital to home proved difficult for them. This study demonstrated the benefits of social support from family and friends, as long as it was considered appropriate and focused on the person’s needs.

## Appendix C

**Table A3:** The intervention studies included in the systematic review.

Author(s), Year	Country of the Study	Subjects	Psychological Construct Assessed	Intervention (If Applicable)	Study Design	Main Results
Bak et al., 2006 [56]	Germany	Sixty-four (48 m, 20 f; 67.3 age) consecutive patients were in treatment in an orthopaedic hospital.	QOL/HRQoL (SF-36), functional independence (FIM).	Yes (rehabilitation: kinesitherapy, prosthesis, occupational therapy-physiotherapy, educational programme, psychological or neuropsychological interventions).	Quantitative (cross-selection).	Both the SF-36 and the FIM were found to be sensitive enough to detect longitudinal changes in QOL/HRQoL and functional independence in the sample studied. The results should be interpreted with caution due to possible biases (floor and ceiling effects).
Horne et al., 2017 [40]	USA	Thirteen (58.3% f 60 age) participants had a LLA with a primary cause of peripheral vascular disease, diabetes, and/or end-stage renal disease at a large academic tertiary care.	Pain (SF-MPQ-2), depression, and anxiety (HADS), intervention (intervention journal card, intervention questionnaire, feasibility questionnaire).	Yes (desensitisation therapy).	Quantitative (pre-experimental repeated measure study).	This study provides some support for the use of tactile desensitisation in the acute postoperative period following lower limb amputation. Participants reported that the intervention helped to reduce pain.
